# Returning a Shelter Dog: The Role of Owner Expectations and Dog Behavior

**DOI:** 10.3390/ani12091053

**Published:** 2022-04-19

**Authors:** Lauren Powell, Brittany Lee, Chelsea L. Reinhard, Margaret Morris, Donya Satriale, James Serpell, Brittany Watson

**Affiliations:** 1 School of Veterinary Medicine, University of Pennsylvania, Philadelphia, PA 19104, USA; balee@vet.upenn.edu (B.L.); creinh@vet.upenn.edu (C.L.R.); serpell@vet.upenn.edu (J.S.); brittawa@vet.upenn.edu (B.W.); 2 Charleston Animal Society, North Charleston, SC 29406, USA; mmorris@charlestonanimalsociety.org (M.M.); dsatriale@charlestonanimalsociety.org (D.S.)

**Keywords:** adoption, animal shelter, behavior, dog, expectations, human–animal bond, ownership responsibility

## Abstract

**Simple Summary:**

Most dog adopters are typically highly satisfied with dog ownership, although a number of adopted dogs are returned to animal shelters following adoption, which can be stressful for the owner and the dog. In this study, we looked at adopters’ expectations for dog ownership prior to adoption and their experience with dog behavior in the first days, weeks, and months following adoption relative to the risk of return. We found owners who returned their dog to the shelter within three months of adoption had higher expectations for their dog to be healthy, exhibit desirable behavior, and for the human–dog bond compared with adopters who did not return their dogs. There were no differences in expectations for ownership responsibilities and challenges between returning and non-returning owners. We also found two-thirds of owners experienced some behavioral problems following adoption, although behaviors such as training difficulty and fear decreased over time. Our findings may be useful for animal shelters to reduce returns by ensuring adopters have realistic expectations about the human–dog relationship and the occurrence of behavioral problems.

**Abstract:**

Millions of animals are adopted from animal shelters in the United States each year, although some are returned post-adoption, which can decrease both the animals’ chances of future adoptions and the owners’ willingness to adopt again. In this study, we investigated the impact of adopter expectations for ownership and animal behavioral problems on post-adoptive dog returns at a large animal shelter in South Carolina. Between June–September 2021, 132 dog adopters completed a survey about their expectations for ownership through Qualtrics. Twenty-nine adopters returned their dogs to the shelter within three months of adoption, with a median length of ownership of eight days. Owners completed follow-up questionnaires about their perceptions of adoption and dog behavior at two days, two weeks, and four months post-adoption. Categorical principal component analysis revealed three factors pertaining to adopters’ expectations for ownership. Independent *t*-tests showed returning owners had significantly higher expectations for dog behavior and health (*t* = −2.32, *p* = 0.02) and the human–dog bond compared with non-returning owners (*t* = −2.36, *p* = 0.02). Expectations for ownership responsibilities did not differ between the groups. Two-thirds of adopters experienced dog behavioral problems post-adoption, although training difficulty decreased significantly between two days and four months (*F* = 5.22, *p* = 0.01) and nonsocial fear decreased between two weeks and four months post-adoption (*X*^2^ = 10.17, *p* = 0.01). Shelters may benefit from utilizing adoption counselling to ensure adopters understand the potential for dog behavioral problems in the early stages of ownership and develop appropriate expectations for the human–dog relationship. Post-adoption behavioral support may also help some owners to overcome behavioral difficulties as their dogs adapt to the new environment.

## 1. Introduction

Dog ownership is common in the United States and globally, with an estimated 471 million dogs kept as pets around the world [1]. Many prospective owners acquire their dogs from animal shelters, as adoption is often considered the most ethical method of dog acquisition [2]. In the United States, approximately four million animals were adopted from animal shelters in 2019 [3]. However, about 15% of all adoptions result in the animal being returned to the shelter [4,5,6,7]. Short-term adoptions can have some benefits, such as providing short-term stress relief for dogs [8], and giving shelter staff an opportunity to learn more about the dogs’ behavior out of the shelter environment. However, a return to the shelter environment can also lead to increased canine stress [9,10,11], and can increase the likelihood of euthanasia, particularly when a dog is returned for behavioral or medical reasons [4]. Returned adoptions can also be stressful for owners [12], and can decrease an owner’s willingness to adopt again following the return [13].

Behavioral problems are a leading cause of returned adoptions. In a recent study by the authors, 36% of dogs returned to a South Carolina shelter were disowned due to behavioral problems and a further 18% due to incompatibility between the adopted dog and the existing pets [4]. At a Texas shelter, 56% of dog returns were due to behavioral issues, most commonly aggression towards humans and other animals [14]. In the UK, three out of five dog returns were attributed to behavioral problems [5] and, in Australia, 22% of returns occurred due to behavioral problems, with a further 12.9% due to problems between the adopted dog and existing pets [15]. Research has also shown that returned dogs are significantly more likely to exhibit behavioral problems than retained dogs [6]. However, each of these studies relied on owner reports of animal behavior, which can be prone to error [16,17]. Other factors, such as owners’ expectations for, and tolerance of, behavioral problems are also likely to impact perceptions of dog behavior and the possibility of return.

Unrealistic expectations for ownership have been documented as a primary return reason in 7 to 13% of dog returns [4,14]. Diesel et al. [5] found owners who underestimated the work and effort involved in dog ownership were 10 times more likely to return their animal post-adoption than owners who overestimated the effort involved. Patronek, et al. [18] also found that owners who said caring for their dog was more work than expected were four times more likely to relinquish the dog. However, prospective dog owners are likely to have a multitude of expectations beyond the amount of effort required for ownership. In an Australian study of 3465 prospective dog owners, 89% of respondents expected increased happiness, 74% expected decreased stress, and 61% expected decreased loneliness from dog ownership [19]. In the same study, 64% of respondents expected increased responsibility, 62% expected challenges with dog training, and 50% expected to face challenges with dog behavioral issues [19]. A Canadian study also found adopters expected health benefits and companionship from ownership, while simultaneously expecting challenges, such as unexpected costs and increased commitment [20]. To our knowledge, only one study to date has considered a range of owner expectations when investigating returned adoptions and found no difference between returning and retaining owners in their expectations for emotional or physical support from dog ownership [21].

The possible interactions between owner expectations and dog behavior have also been neglected in most research examining returned adoptions. In the Patronek et al. [18] study described above, owners who underestimated the work involved in caring for their dog were also more likely to report undesirable behaviors, such as barking, chewing, and aggression [18], which raises the question: were the relinquishments attributable to unrealistic owner expectations, undesirable dog behavior, or a combination of the two? The goal of this study was to examine the role of adopters’ expectations and dog behavior in the retention of newly adopted dogs.

## 2. Materials and Methods

### 2.1. Animal Shelter

Participants were recruited from Charleston Animal Society between 8 June and 30 September 2021. Charleston Animal Society is an open admission shelter in South Carolina, USA, that had an intake of 4473 cats and 2815 dogs in 2021 [22]. During the study period, 560 dogs were adopted from the shelter. Charleston Animal Society employs an open adoption policy to develop a non-judgement relationship with their adopters and reduce barriers to adoption. Prior to adoption, all potential adopters meet with shelter staff for adoption counselling, during which staff will describe the adoption process and engage in conversation to help guide the adopter to choose a pet that meets their criteria. Shelter staff also provide adopters with all the available information about the pet, including known medical and behavioral concerns, as well as information about basic pet care and resources to help the adopter and the pet successfully transition into their home. Charleston Animal Society encourages adopters to return their animals to the shelter if the adoption is not suitable and provides a refund voucher to put towards future adoptions if the return occurs within 30 days of adoption. Adopters can also access post-adoption behavioral support through the shelter’s behavioral team and a free veterinary appointment for their adopted animal at a local veterinary clinic. Individuals were eligible to participate in this study if they were over the age of 18 years and planned to be the primary caregiver of the dog they were adopting. The study was reviewed and approved by the University of Pennsylvania Institutional Review Board (Philadelphia, PA, USA, protocol number 849107).

### 2.2. Study Protocol

Participants were informed of the requirements of the study and provided written consent at the shelter prior to completing the first survey through Qualtrics on an iPad. The survey was completed after participants had selected their dog and completed adoption counselling with a shelter staff member. Participants were informed that their individual responses were confidential, would not be directly shared with the animal shelter staff, and would not impact their adoption in any way. If participants returned their dog to the shelter, they could choose to allow researchers to share their responses to the Canine Behavioral Assessment and Research Questionnaire (C-BARQ) with the shelter staff. We included the opportunity for data sharing to provide returning owners with a mechanism through which they could help shelter staff to better understand the animal’s behavior in the home environment and to identify appropriate successive adopters. Ultimately, we were not asked by the shelter to share C-BARQ data for any returned dogs.

Researchers then contacted participants via email two days, two weeks, and four months after the adoption to complete follow-up surveys through Qualtrics. These intervals were selected based on our previous research that showed about 25% of returns occurred within two days, about 80% occurred within two weeks and almost all returns occurred prior to four months [4,13]. Participants who did not respond to the two-day and two-week surveys within 24 h were sent an email reminder. If the participants still failed to respond, a researcher phoned the participant 48 h later. Participants who did not respond to the four-month survey within 48 h were sent a follow-up email. Six participants completed the two-day survey, and three participants completed the two-week survey by phone.

Adopter’s responses at each timepoint were linked using the dog’s shelter ID number. Researchers also used the dog’s ID number to record the animal’s details (age at adoption, sex, weight, breed, intake type, and length of stay) from the shelter’s data management software (PetPoint Data Management System, Version 5, Pethealth Software Solutions Inc., Oakville, ON, Canada). Dog breed was recorded by shelter staff at intake based on the dog’s phenotypic characteristics or the breed provided by the relinquishing owner. Primary breed was then categorized based on the American Kennel Club’s breed groupings [23], with an additional category for Pit Bull-type breeds [24]. Age was categorized as puppy (<6 months), young adult (>6 months–2 years), adult (>2 years–8 years), and senior (>8 years) [4], and size was categorized as small (0–19 lbs), medium (20–59 lbs), and large (≥60 lbs) [14]. All animals were sterilized prior to adoption.

The dog’s ID number was also used to track the animal’s return status in the PetPoint software. Animals that re-entered the shelter within three months of adoption were coded as returned adoptions. Researchers documented the animal’s return reasons from the PetPoint software. Return reasons were entered by animal shelter staff at the time of return based on the returning owner’s reports. Adopters were not contacted to complete the follow-up surveys if they had already returned the animal.

### 2.3. Baseline Questionnaire

The baseline questionnaire, completed at the time of adoption, included several demographic questions about the adopter’s gender, age, and previous dog ownership history. Participants were then asked to indicate how much they agreed with 36 statements about their expectations for ownership. Possible answers ranged from strongly disagree (1) to strongly agree (5). Most of the statements were based on a previous questionnaire developed by O’Connor et al. [20] to investigate owner expectations for adoption in Canada, although several items were adapted to better reflect the C-BARQ, which was used to measure canine behavior post-adoption (described further below).

### 2.4. Follow-Up Questionnaires

The two-day, two-week, and four-month follow-up surveys included a series of five-point Likert-scale questions about the owner’s perceptions of: (1) how well their dog has adapted to the home, with possible answers ranging from “not at all” to “extremely well”; (2) what extent their expectations for ownership had matched their experience so far, with possible answers ranging from “owning this dog has been much more difficult than I expected” to “owning this dog has been much easier than I expected”; (3) how satisfied they are with their adopted dog, with possible answers from “very dissatisfied” to “very satisfied”. Owners were also asked if they were experiencing any problems with their dog’s behavior. Possible responses included “no problems”, “only minor problems”, “moderate problems”, or “serious problems”.

Owners were then asked to complete the mini C-BARQ, which is a valid and reliable tool for measuring dog behavior [25]. The mini C-BARQ includes 42 items that form 14 subscales and 9 miscellaneous items. In each item, owners are asked to report the frequency or severity of their dog’s behavior in response to a specific circumstance or stimulus in the recent past on a five-point scale from never/no signs (0) to always/extreme signs (4). If an adopter had not observed their dog in the described situation, they could select “N/A” and the data were coded as missing. C-BARQ subscale scores were calculated by summing and averaging the item scores in each subscale. Two items in the “training difficulty” subscale were reverse scored prior to summation, so that higher scores were indicative of less desirable behavior across all subscales and miscellaneous items. C-BARQ data were excluded for dogs below six months of age as the C-BARQ has not yet been validated for use in puppies [25]. The C-BARQ subscale for touch sensitivity, which measures the dog’s response to bathing and grooming, was also excluded due to a high proportion of missing values.

### 2.5. Categorical Principal Component Analysis (CATPCA)

To reduce the number of variables relating to adopters’ expectations for ownership, we used categorical principal component analysis (CATPCA) with ordinal variables to group the 36 expectations. Two cases were removed based on visual inspection of the component score plots. Scores that fell outside the −3.5 to +3.5 range were considered outliers (*n* = 2). All items were retained in the final solution as each had a Variance Accounted For (VAF) value ≥0.25 [26] and a factor loading >0.30. Three factors were retained based on visual inspection of the scree plots. A rotated solution was applied to aid interpretability. Items that loaded onto more than one factor were assigned to the factor with the highest loading. Factor scores were then calculated for each participant by summing and averaging the items within each factor.

### 2.6. Statistical Analysis

Descriptive statistics were calculated, and data were assessed for normality using Shapiro–Wilk tests and visual inspection of histograms. To compare returning and non-returning owners’ expectations for ownership, we used Mann–Whitney U tests for individual items and independent *t*-tests for CATPCA factor scores. A Mann–Whitney U test was also used to compare length of ownership between animals returned for animal-related reasons and owner-related reasons. For C-BARQ subscales with normal distributions, we conducted linear mixed models to assess changes in subscale scores including timepoint as a fixed variable (two days, two weeks, and four months) and dog ID as a random variable. For non-normally distributed C-BARQ scales, we ran Friedman tests to investigate changes in scores across each timepoint. To maximize the use of available data, we also ran Wilcoxon signed-rank tests to compare changes in C-BARQ subscale scores between two days and two weeks, two weeks and four months, and two days and four months post-adoption. Non-normal C-BARQ subscales and miscellaneous items were also categorized as binary variables (score of 0/score > 0) to describe the prevalence of each behavior, as has been performed previously [25]. Statistical analyses were conducted in IBM SPSS Statistics for Windows Version 27.0, and *p*-values < 0.05 were considered statistically significant. We made an a priori decision not to adjust p-values for multiple testing as the study was exploratory, we were interested in the statistical significance of the individual tests, and the analyses were based on a priori hypotheses [27].

## 3. Results

### 3.1. Study Response Rates

The baseline survey was completed by 135 dog adopters, although three prospective adopters did not adopt a dog at the time of survey completion and were subsequently removed from the study. A total of 29 dogs were returned to the shelter, 10 of whom were returned within two days and were excluded from the two-day post-adoption survey. Fifty-eight adopters completed the two-day post-adoption survey, eight of whom later returned their dogs. The two-week post-adoption survey was completed by 38 adopters, three of whom later returned their dogs, and the four-month survey was completed by 28 adopters. Data were excluded from the two-day survey if the participants did not respond within three days (*n* = 1 returned dog, *n* = 2 non-returned dogs), from the two-week survey if participants did not respond within one week (*n* = 1 non-returned dog), and from the four-month survey if participants did not respond within two weeks (*n* = 1 non-returned dog).

### 3.2. Demographics

The sample included 69 female adopters and 63 male adopters, most of whom were current or previous dog owners (Table 1). The adopted dogs were primarily male, young adults or adults, and medium-sized. Pit Bull-type breeds (*n* = 61, 46.2%), hounds (*n* = 27, 20.5%), and sporting breeds (*n* = 19, 14.4%), such as Labrador Retrievers, were the most represented breeds (Supplementary Table S1).

Of the 132 adopted dogs, 29 (22%) were returned to the shelter within three months of adoption, with a median length of ownership of 8.0 days (IQR 2.0–21.0 days). More than half of the animals were returned for animal-based reasons, particularly related to animal behavior (58.6%), while 34.5% were returned for owner-related reasons (Table 2). Returns for animal-related reasons had a significantly shorter median length of ownership of 2.5 days (IQR 1.0–12.0) compared with owner-related returns with a median length of ownership of 8.0 days (IQR 5.0–38.0, *U* = 129.50, *Z* = 2.05, *p* = 0.04). There were no significant differences between returning and non-returning owners in gender (*X*^2^ = 0.83, *p* = 0.41), age (*X*^2^ = 1.76, *p* = 0.90), or previous ownership history (*X*^2^ = 5.75, *p* = 0.09, Table 1). Animals who were returned did not differ from those who were not returned in age (*X*^2^ = 4.02, *p* = 0.27), sex (*X*^2^ = 0.18, *p* = 0.42), size (*X*^2^ = 1.75, *p* = 0.48), intake type (*X*^2^ = 3.36, *p* = 0.50), previous return status (*X*^2^ = 0.03, *p* = 0.87), length of stay (*U* = 1208.50, *Z* = −1.57, *p* = 0.12, Table 1), or breed group (*X*^2^ = 5.87, *p* = 0.40, Supplementary Table S1).

### 3.3. Expectations for Ownership

The three CATPCA factors explained 25.6%, 15.6%, and 8.0% of the variance, respectively. The total eigenvalues were 9.22, 5.62, and 2.89. The three factors described dog behavior and physical health, the human–dog bond, and owner responsibilities.

**Figure 1:**
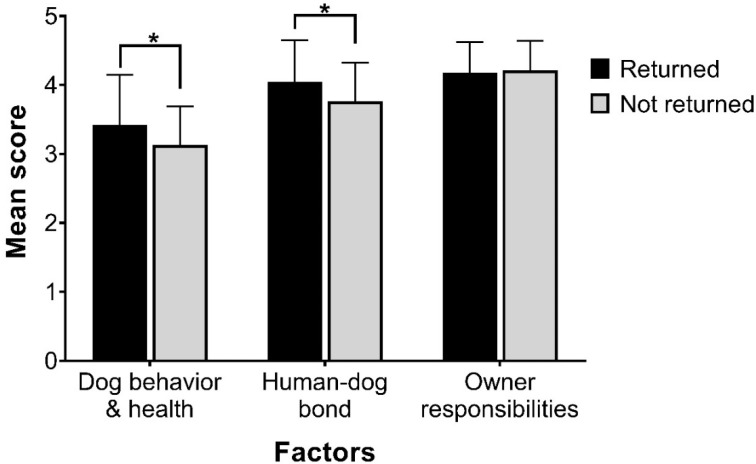
Mean factor scores ± standard deviation for owner expectations. * Indicates there was a statistically significant difference between return and non-return owners (*p* < 0.05).

Returning owners had significantly higher expectations for dog behavior and physical health (*t* = −2.32, *p* = 0.02), and the human–dog bond (*t* = −2.36, *p* = 0.02), but not owner responsibilities (*t* = 0.33, *p* = 0.74) compared with non-returning owners (Figure 1). Returning owners were more likely to expect their dog not to be fearful in new situations, to be friendly towards children, to be responsive to training, and not to dig or chew inappropriate objects (Table 3). Returning owners also had significantly higher expectations for their dog to be healthy when they brought him/her home from the shelter, to be a form of emotional support, to be sensitive to their feelings, and to be excited to greet them when they come home.

### 3.4. Owners’ Perceptions of Adoption

**Figure 2:**
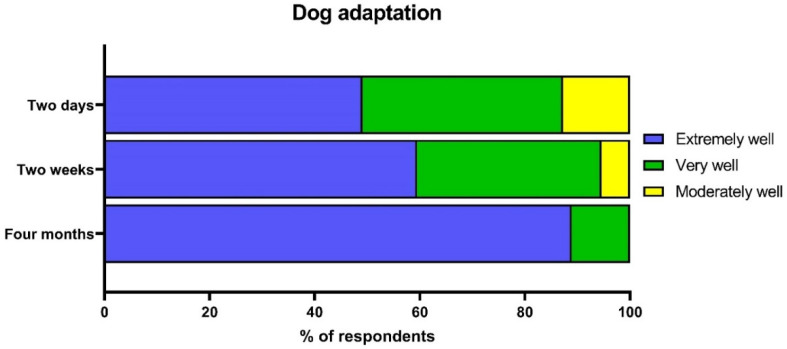
Owners’ perceptions of their dog’s adaptation to the home at two days, two weeks, and four months post-adoption.

Owners’ perceptions of how well their dog has adapted to their home are shown in Figure 2. Between two days, two weeks, and four months, the proportion of owners who said their dog had adapted extremely well increased, while the proportion who said their dog had adapted very well or moderately well decreased. Among the seven returning owners with data at two days (i.e., adopters who still owned the dog two days after adoption but returned them at a later date), four said their dog had adapted extremely well, one said their dog had adapted very well, and two said their dog had adapted to their home moderately well after two days. Of the three return owners with data at two weeks (i.e., adopters who owned the dog two weeks post-adoption but later returned them), two said their dog had adapted very well and one said their dog had adapted moderately well. The vast majority of owners were also very satisfied with their dog including 87.3% of owners at two days (*n* = 48 out of 55), 91.9% at two weeks (*n* = 34 out of 37), and 100% at four months (*n* = 27).

**Figure 3:**
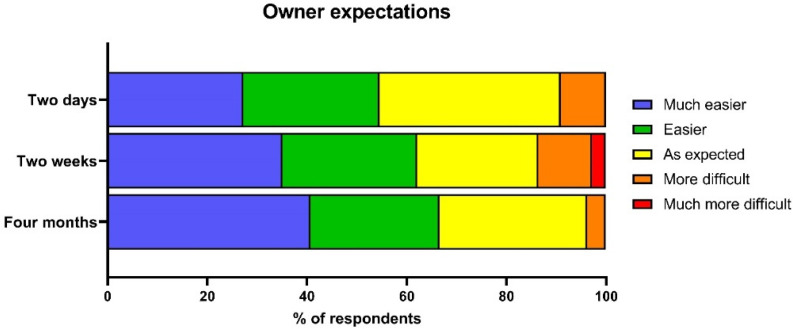
Owner expectations relative to their expectation of adoption at two days, two weeks, and four months post-adoption.

Figure 3 shows adopters’ expectations for ownership relative to their experience at each time point. Approximately half of the adopters said that owning the dog had been easier than they had expected at two days, which increased to 62.1% at two weeks and 66.6% at four months. A much smaller proportion of adopters found ownership more difficult than expected. At two days, five owners said that owning the dog was more difficult than they had expected (9.2%), three of whom later returned their dogs to the shelter. At two weeks, five owners also found ownership more difficult than expected (13.5%), although only one of those owners later returned their dog to the shelter. Finally, at four months, only one owner said that owning the dog was more difficult than they had expected (3.7%).

### 3.5. Dog Behavior Post-Adoption

About a third of the owners said they were not experiencing problems with their dog’s behavior two days after adoption (*n* = 16, 29.1%), although two-thirds said they experienced minor problems (*n* = 36, 65.5%), and 5.5% said they were experiencing moderate problems (*n* = 3). Owner responses were similar two weeks post-adoption, with 32.4% of owners indicating they were not experiencing any problems with their dog’s behavior (*n* = 12), 59.5% experiencing minor problems (*n* = 22), and 8.1% experiencing moderate behavioral problems (*n* = 3). At four months, 33.3% of owners said they were not experiencing any problems with their dog’s behavior (*n* = 9) and 66.7% were experiencing only minor problems (*n* = 18).

Table 4 shows the occurrence of behavioral problems based on C-BARQ scores. Nonsocial fear and separation-related behavior were the most prevalent behaviors across each time point. Dog-directed fear and chewing inappropriate objects were also relatively common, while owner-directed aggression and familiar dog aggression were comparatively rare.

Table 5 displays the changes in normally distributed C-BARQ subscales and miscellaneous items from two days to four months post-adoption. Linear mixed models showed training difficulty decreased significantly between two days and two weeks (mean difference = −0.51, 95% CI −0.97, −0.05), and between two days and four months post-adoption (mean difference = −0.78, 95% CI −1.26, −0.29). Excitability appeared to increase following adoption, although the difference was not statistically significant (*p* = 0.06). There were no statistically significant differences in chasing, energy levels, or pulling on the leash between two days and four months post-adoption (Table 5).

The non-normally distributed C-BARQ subscales and items are shown in Table 6. Nonsocial fear decreased significantly following adoption, with post hoc analyses revealing a significant reduction between two weeks and four months post-adoption. Dog-directed aggression appeared to increase post-adoption, although post hoc analyses showed no significant differences across the three time points. Chewing inappropriate objects increased between two days and two weeks post-adoption, although there was no significant difference when considering all three time points.

## 4. Discussion

The goal of this study was to elucidate the role of owner expectations and dog behavior in the retention of newly adopted dogs. We found 22% of adopted dogs were returned within three months of adoption, which exceeded the previous rate of 16% from the same shelter between 2015 and 2019 [4] and the overall dog return rate of 13% in 2021 [22], possibly indicating that returning owners were more interested in reporting their expectations for ownership at the time of adoption than non-returning owners. Almost two-thirds of returned animals were disowned due to animal behavior, confirming the growing body of evidence that cites behavior as a common reason for return [5,7,14]. Approximately one-third were returned for owner-based reasons, such as housing issues or a lack of time, which also mirrors some previous data [14].

We found several differences between return and non-return adopters’ expectations for ownership. Firstly, returning owners had higher overall expectations for dog behavior, and were more likely to expect their dog to be friendly towards children, not to be fearful in new situations, and not to be destructive. Concerns about animal behavior are common among prospective adopters [19,28], typically related to aggression, incompatibility with current pets, fearfulness, destructive, or vocal behaviors [28]. However, to our knowledge, this is the first time that returning owners have been shown to report higher overall expectations for desirable animal behavior prior to bringing the animal home. Given these expectations, returning owners may have been less satisfied with their animal’s behavior, regardless of whether the behavior was appropriate or not. Such high expectations could have detrimental effects on the development of the human–dog bond and the strength of owner attachment [29,30]. Previous research has found that owners who are dissatisfied with their animal’s behavior often report weaker levels of attachment to the pet [29], whereas owners who share similar personality traits with their pets typically report more positive perceptions of their animal’s behavior and increased human–animal attachment [31].

Secondly, return owners were more likely to expect their dog to be healthy following adoption. A previous study of prospective dog owners found 60% of adopters considered it extremely important that their dog was physically healthy at adoption [32]. This expectation is somewhat at odds with current evidence that shows 50% of adopted dogs exhibit a health problem within the first two weeks of ownership [33,34]. There were no dogs returned for medical reasons in this study, possibly due to the provision of free veterinary appointments post-adoption, but the disconnect between owners’ expectations and the occurrence of health problems highlights a need for further education to ensure prospective owners develop appropriate expectations.

Thirdly, we found that returning owners had higher expectations for the human–dog bond compared with non-return owners and were more likely to expect their dog to provide emotional support, to be sensitive to their emotions, to be responsive to training, and to be excited to greet them. It is possible that some returning owners underestimated the time necessary for dogs to adjust to their new home and develop a strong relationship with their owner, and thus may have been dissatisfied with ownership and more likely to return the animal. Supporting this theory, previous research has shown that owners perceive more advantages of pet ownership 18 months after they acquire their pet compared with 6 months post-acquisition, including benefits such as providing companionship, having someone to talk to, and experiencing more liveliness around the home [35]. Alternatively, some owners may have overestimated the emotional benefits that are attainable from pet ownership. Some studies have shown dog acquisition improves human mental wellbeing [36,37,38], but other studies have found null effects [39,40,41] or even a detrimental association between pet ownership and mental health [42]. Despite the uncertainty in the scientific literature, extensive media coverage has led to a widespread belief that pet ownership is beneficial for mental health [43]. A 2016 survey by the Human Animal Bond Research Institute found that 71% of pet owners were aware of scientific evidence that showed pet ownership can improve human health [44], and studies have shown that most prospective dog owners expect their dog to improve their mental and physical health [19]. The positive media coverage of pet ownership was especially evident during the COVID-19 pandemic, at a time when global interest in pet ownership spiked [45]. Future studies investigating the relationship between owners’ expectations for health benefits prior to ownership and their realized health benefits post-adoption would help to elucidate the relationship between owner expectations, dog retention, and health outcomes. Nonetheless, shelters should aim to help adopters develop appropriate expectations for the human–animal relationship by providing balanced information about the current state of the evidence regarding dog ownership and emotional wellbeing.

Returning owners did not differ from non-returning owners in their expectations regarding the effort required in dog ownership. Both returning and non-returning owners strongly agreed that they’d have to walk, train, and play with their new dog, and that they would need to take an active role to help their new dog adjust to its environment. Owners also agreed that they could face unexpected financial costs from ownership and that they may have to make changes to their schedule to accommodate their new dog. Our findings mirror those of O’Connor et al. [20] who also found adopters expected to take an active role helping their new pet adjust to their home and acknowledged the potential for unexpected costs from ownership. The results are at odds with the findings of two previous studies that showed relinquishing owners were more likely to underestimate the effort required in ownership [5,18]. Adopters in this study may have developed appropriate expectations for ownership responsibilities following adoption counselling, as adopters met their dog and discussed its needs with shelter staff prior to completing the expectations survey.

Considering dog behavior, we found two-thirds of owners said they were experiencing some problems with their dog’s behavior at two days, two weeks, and four months post-adoption, and a relatively high proportion of dogs exhibited undesirable behavior based on the C-BARQ scores. Previous studies have also found behavioral problems are relatively common among newly adopted dogs [6,33,46]. Unfortunately, the short median length of ownership among returned dogs and the small sample size meant we were unable to compare dog behavior between returned and non-returned dogs. It is therefore unclear whether the severity of behavioral problems differed between returned and non-returned dogs, or whether returning and non-returning owners simply differed in their expectations for and tolerance of behavioral problems. The use of pre-adoption behavioral assessments in the shelter (including relinquishing owner reports, staff observations, daily behavioral notes, and/or standardized assessments) may also have reduced the severity of undesirable behaviors among adopted dogs, as dogs with extreme behavioral issues were likely identified prior to adoption and subjected to increased behavioral modification. Even so, the frequency of behavioral problems among both returned and non-returned dogs and the high proportion of behavioral returns supports the use of behavioral counselling at the time of adoption and the provision of post-adoption behavioral support. Future research investigating the utilization and efficacy of behavioral support in reducing returns would be of benefit to the field.

Some aspects of dog behavior also improved over the first four months post-adoption. Training difficulties decreased significantly between two days and four months post-adoption, suggesting that dogs were more likely to obey a “sit” or “stay” command and were less distracted by interesting sights, sounds, and smells. Nonsocial fear also decreased from two weeks to four months, indicating dogs exhibited less fearfulness in response to loud noises, unfamiliar objects, and situations. Interestingly, these behavioral changes mirror some of the differences in owner expectations described above (i.e., the higher expectation for trainability and the decreased expectation for fearfulness among returning owners). It is possible that poor trainability and nonsocial fear are common behaviors among newly adopted dogs that subside over time as the animal adapts to the new home and develops a bond with their new owner. Given the short length of ownership, most returning owners were unlikely to observe these behavioral improvements before they returned the animal. We also found some evidence that the occurrence of destructive chewing increased between two days and two weeks post-adoption, although there was no significant difference across the three time points. Adjusting owners’ expectations for the potential for undesirable behaviors in the early stages of ownership, particularly training difficulties and nonsocial fear, and the length of time needed to observe behavioral improvements may help to reduce return rates.

It is important to note that most owners with follow-up data said they were very satisfied with their new dog, that their dog had adapted to their home extremely or very well, and that their expectations for ownership had matched the experience or that owning the dog had been easier than they’d anticipated. The high levels of ownership satisfaction irrespective of the prevalence of behavioral problems suggests that, for most owners, their experience with canine behavioral problems did not exceed their expectations for behavioral challenges and did not impact the development of the human–dog bond. While we were unable to statistically compare satisfaction between return and non-return owners, we did not see a clear pattern between ownership satisfaction and return status. However, animals that were returned for animal-related reasons had a significantly shorter length of ownership, so many of the returns with follow-up data were attributable to owner-related reasons, which could differentially impact ownership satisfaction. For example, it is plausible that behavioral problems may have a more detrimental impact on the human–animal relationship and ownership satisfaction than cases where animals are returned due to changes in their owner’s circumstances.

This study has provided preliminary insights into the complex relationship between owner expectations, dog behavior, and the retention of adopted dogs, although the findings must be considered in the light of several limitations. The sample size was relatively small, which impeded detailed statistical analyses between the returned and non-returned groups. The small sample size may also have increased the likelihood of Type II error. For example, we did not find any significant differences in the characteristics of returned and non-returned dogs, despite previous research showing an association between the risk of return and dog characteristics, such as size and age [4,5]. As a result of the small sample size and the associated risk of type II error, we did not include adjustment for multiple testing, which can further increase the risk of type II error. However, the lack of multiple testing correction may have led to an inflated risk of type I error (i.e., identifying false positive results). It is possible that including multiple testing correction may have altered the results, so further studies are needed to confirm our findings. The results may also be affected by non-response bias. Participation was voluntary, so it is possible that some adopters were more willing to participate than others and that the sample may not be representative of all adopters. For instance, the return rate in this sample was higher than the overall return rate at the shelter during the study period, suggesting that return owners may have been more willing to share their expectations and experiences regarding dog ownership. It is also possible that participation in the study itself prompted owners to critically appraise their dog’s behavior or their relationship with their dog, which could have also increased the likelihood of return. As shelter staff distributed the baseline survey to participants, we were unable to ascertain a response rate. A considerable portion of participants also failed to respond to the follow-up surveys (52% at two days, 66% at two weeks, and 73% at four months). It is again possible that these participants systematically differed from participants who did respond. For example, owners who were very satisfied with their new dog may have been more motivated to respond to the follow-up surveys than owners who were only moderately satisfied or dissatisfied. Conversely, owners of dogs with behavioral problems may have been more inclined to complete the survey due to their challenges with animal behavior. A small number of owners preferred to complete the follow-up surveys over the phone, which may have introduced social desirability bias, although this seems unlikely given all owners had the option to answer the follow-up surveys confidentially via Qualtrics. The return reasons were based on owners’ reports, which may be prone to error [47]. For example, it is not clear whether dogs who were returned for animal behavior were exhibiting true behavioral disorders or whether they were simply displaying behaviors that the owners deemed undesirable. The results are also representative of a single shelter and it is not clear whether adopters from other shelters would have the similar expectations for ownership or perceptions of adoption. Studies including more study sites and a longer follow-up period could also uncover further differences in owner expectations between retained dogs, short-term returns, and owner relinquishments.

## 5. Conclusions

In conclusion, we found most newly adopted dogs displayed some undesirable behaviors in the early days, weeks, and months following adoption, although several behaviors, such as training difficulty and nonsocial fear, improved during this time. Congruent with the current body of evidence, most post-adoption returns were attributable to animal behavioral issues. However, we also found that returning owners had significantly higher expectations for desirable animal behavior and the development of the human–dog bond compared with non-return owners. Expectations for ownership responsibilities did not differ between the groups. Our findings support the ongoing use of adoption counselling to ensure adopters develop appropriate expectations for the human–animal relationship and the likely occurrence of undesirable behaviors in the first four months post-adoption. The provision of post-adoption support services may also assist some owners to overcome behavioral challenges.

## Figures and Tables

**Table 1:** Study characteristics of owners and adopted dogs (*n* = 132).

	Not Returned % (*n*)	Returned % (*n*)	Total % (*n*)
***Owner***			
**Gender**			
Male	45.6 (47)	55.2 (16)	47.7 (63)
Female	54.4 (56)	44.8 (13)	52.3 (69)
**Age**			
18–29	35.0 (36)	37.9 (11)	35.6 (47)
30–39	22.3 (23)	27.6 (8)	23.5 (31)
40–49	13.6 (14)	13.8 (4)	13.6 (18)
50–59	15.5 (16)	10.3 (3)	14.4 (19)
60–69	8.7 (9)	3.4 (1)	7.6 (10)
70+	4.9 (5)	6.9 (2)	5.3 (7)
**Dog ownership history**			
Current	50.5 (52)	34.5 (10)	47.0 (62)
Previous	46.6 (48)	55.2 (16)	48.5 (64)
Never	2.9 (3)	10.3 (3)	4.5 (6)
***Dog***			
**Sex**			
Male	61.2 (63)	65.5 (19)	62.1 (82)
Female	38.8 (40)	34.5 (10)	37.9 (50)
**Age**			
Puppy (≤6 months)	20.4 (21)	20.7 (6)	20.5 (27)
Young adult (>6 months−2 years)	30.1 (31)	44.8 (13)	33.3 (44)
Adult (>2–8 years)	41.7 (43)	34.5 (10)	40.2 (53)
Senior (>8 years)	7.8 (8)	0.0 (0)	6.1 (8)
**Size ^a^**			
Small (<19 lbs)	11.7 (12)	6.9 (2)	10.6 (14)
Medium (>19–<59 lbs)	70.9 (73)	65.5 (19)	69.7 (92)
Large (>59 lbs)	17.5 (18)	27.6 (8)	19.7 (26)
**Intake type**			
Owner surrender	13.6 (14)	24.1 (7)	15.9 (21)
Return	8.7 (9)	13.8 (4)	9.8 (13)
Seized/custody	20.4 (21)	17.2 (5)	19.7 (26)
Stray	36.9 (38)	24.1 (7)	34.1 (45)
Transfer in	20.4 (21)	20.7 (6)	20.5 (27)
**Previous Return**			
Yes	12.6 (13)	13.8 (4)	12.9 (17)
No	87.4 (90)	86.2 (25)	87.1 (115)
**Length of stay (days) ^b^**	12.0 (8.0–19.0)	11.0 (6.5–16.5)	12.0 (8.0–18.8)

^a^ Based on weight (lbs) at time of adoption; ^b^ data based on length of stay in the shelter presented as median (IQR).

**Table 2:** Return reasons (*n* = 29).

Return Reason	*n*	%
**Animal-based reasons**	**17**	**58.6**
Chases animals	1	3.4
Doesn’t like other pets	3	10.3
Escapes	2	6.9
Multiple behavior issues	1	3.4
Pets in home didn’t like	4	13.8
Too active	3	10.3
Too much energy	1	3.4
Too noisy	1	3.4
Requires too much exercise	1	3.4
**Owner-based reasons**	**10**	**34.5**
Change in lifestyle	1	3.4
Homeless	1	3.4
Inadequate housing/yard	1	3.4
Landlord issues	1	3.4
Not enough time	4	13.8
Travel	1	3.4
Unwanted	1	3.4
**Other/Unspecified**	**2**	**6.9**
**Total**	**29**	**100.0**

**Table 3:** Expectations by return status.

Expectations	Not Returned (*n* = 103)	Returned (*n* = 29)	Mann–Whitney U	*p*
***Factor 1—Dog behavior and physical health***				
not to dig or chew inappropriate objects	**3 (2–4)**	**4 (3–4.5)**	**1900.50**	**0.02**
not to escape or run away	4 (3–4)	4 (3–5)	1711.50	0.21
not to chase wildlife	3 (2–3)	3 (2–4)	1804.00	0.07
not to be fearful in new situations	**3 (2–3)**	**3 (2–4)**	**1933.00**	**0.01**
not to be fearful of new people	3 (2–4)	3 (2–4)	1769.50	0.11
not to bark or howl frequently	3 (3–4)	3 (2–4)	1852.00	0.65
to be comfortable being left alone for up to 8 h out of a crate (e.g., during the workday)	3 (2–4)	3 (3–4)	1817.50	0.06
to be comfortable being left alone for up to 8 h in a crate (e.g., during the workday)	3 (2–4)	3 (2–4)	1665.50	0.33
not to growl or bark at me and my family	4 (3–4)	4 (3–5)	1679.50	0.28
to be friendly towards children	**4 (3–4)**	**4 (3.5–5)**	**1943.50**	**0.01**
to have the same personality at home as at the shelter	3 (2–4)	4 (2.5–4)	1799.50	0.08
to already be housetrained	3 (2–3)	3 (3–3)	1639.00	0.39
to be friendly towards other animals and pets	4 (3–4)	4 (3–5)	1773.00	0.10
not to growl or bark at strangers	3 (2–3)	3 (2–4)	1713.00	0.21
to be independent	3 (2–3)	3 (2–3)	1548.50	0.74
to be healthy when I bring them home from the shelter	**4 (3–5)**	**4 (4–5)**	**1926.00**	**0.01**
***Factor 2—Human–dog bond***				
to respond to training	**4 (3–4)**	**4 (4–5)**	**1866.50**	**0.02**
to reduce my stress	4 (3–4)	4 (3–5)	1687.00	0.26
will benefit my health	4 (4–5)	4 (4–5)	1812.00	0.06
to be excited to see me when I come home	**4 (4–4)**	**4 (4–5)**	**1903.50**	**0.01**
to be a form of emotional support	**4 (3–4)**	**4 (4–5)**	**1866.00**	**0.03**
to be sensitive to how I’m feeling	**3 (3–4)**	**4 (3–5)**	**1878.50**	**0.02**
will make me feel safe	4 (3–4)	4 (3–5)	1788.50	0.09
to be my companion	4 (4–5)	4 (4–5)	1658.50	0.31
will be active, energetic, and always on the go	3 (3–4)	4 (3–4)	1738.00	0.14
will be excitable (e.g., before going on a walk)	4 (3–4)	4 (4–5)	1773.50	0.09
***Factor 3—Owner responsibilities***				
I will have to walk my new dog	5 (5–5)	5 (4.5–5)	1477.00	0.90
I will have to train my new dog	5 (5–5)	5 (4–5)	1464.00	0.83
I will have to play with my new dog	5 (5–5)	5 (4.5–5)	1441.00	0.68
there may be unexpected costs associated with my new dog	4 (4–5)	4 (4–5)	1392.00	0.53
to take an active role in helping my new dog adjust to its surroundings	5 (4–5)	5 (4–5)	1572.00	0.59
to consider my new dog part of my family	5 (5–5)	5 (5–5)	1450.50	0.70
taking care of my new dog will require me to learn new skills and information	4 (3–5)	4 (3–5)	1589.00	0.58
bringing a new dog home will be challenging	3 (2–4)	3 (2–3)	1287.00	0.24
I will have to make changes to my schedule to accommodate my new dog	4 (3–4)	4 (3–5)	1534.00	0.82
to be low maintenance ^a^	2 (2–3)	3 (2–3.5)	1711.50	0.20

Bold text indicates there was a statistically significant difference between return and non-return owners (*p* < 0.05). Possible answers for each item ranged from 1 (strongly disagree) to 5 (strongly agree). ^a^ This item was reverse scored in the summation of the factor score as it negatively loaded onto the factor.

**Table 4:** Prevalence of undesirable behavior based on C-BARQ subscales and miscellaneous items.

	2 Days		2 Weeks		4 Months	
	*n*	%	*n*	%	*n*	%
**C-BARQ subscales**						
Stranger-directed aggression	16	44.4	12	40.0	15	55.6
Owner-directed aggression	2	8.7	3	10.3	6	24.0
Dog-directed aggression	16	40.0	13	43.3	17	68.0
Familiar dog aggression	4	14.3	9	37.5	7	33.3
Stranger-directed fear	17	37.8	10	28.6	9	34.6
Nonsocial fear	35	76.1	30	85.7	23	85.2
Dog-directed fear	18	60.0	16	53.3	14	58.3
Separation-related behavior	38	79.2	30	83.3	23	85.2
**C-BARQ miscellaneous items**						
Escapes	24	52.2	13	41.9	14	58.3
Chews inappropriate objects	30	52.6	26	68.4	19	70.4
Urinates against objects in home	18	31.6	14	36.8	6	22.2
Urinates when left alone	17	34.7	13	35.1	8	29.6
Defecates when left alone	10	20.8	10	27.0	7	25.9
Hyperactive, restless, has trouble settling down	34	58.6	19	50.0	16	59.3
Chases tail/hind end	16	28.1	15	39.5	9	33.3
Barks persistently when alarmed or excited	26	46.4	22	59.5	14	51.9

Data were treated as a binary variable (score of 0/score greater than 0).

**Table 5:** Linear mixed models showing change in normally distributed C-BARQ subscale scores and miscellaneous items following adoption.

	F	*p* Value	2 Day		2 Week		4 Month	
			*n*	EMM (95% CI)	*n*	EMM (95% CI)	*n*	EMM (95% CI)
**C-BARQ subscale**								
Excitability	3.07	0.06	38	1.82 (1.50,2.14)	31	2.07 (1.72,2.41)	24	2.40 (2.04,2.75)
Training difficulty	**5.22**	**0.01**	31	2.40 (2.02,2.78)	31	1.89 (1.61,2.17)	22	1.62 (1.30,1.95)
Chasing	0.63	0.54	29	1.76 (1.25,2.27)	26	1.50 (1.04,1.96)	23	1.89 (1.27,2.51)
Energy	0.01	0.99	45	2.28 (1.92,2.64)	34	2.28 (1.88,2.68)	24	2.29 (1.91,2.68)
**C-BARQ miscellaneous items**								
Pulls excessively hard on leash	0.31	0.74	44	1.32 (0.99,1.65)	34	1.47 (1.08,1.86)	24	1.50 (1.09,1.91)

Data are shown as estimated marginal means (95% confidence interval). Bold text shows a statistically significant difference based on timepoint (*p* < 0.05).

**Table 6:** Non-parametric tests showing change in non-normally distributed C-BARQ subscales and miscellaneous items following adoption.

	All Time Points ^a^			2 Days to 2 Weeks ^b^			2 Weeks to 4 Months ^b^			2 Days to 4 Months ^b^		
	*n*	*X* ^2^	*p*	*n*	*Z*	*p*	*n*	*Z*	*p*	*n*	*Z*	*p*
**C-BARQ subscale**												
Stranger-directed aggression	8	0.40	0.82	12	0.33	0.74	15	0.14	0.89	13	−0.16	0.88
Owner-directed aggression	13	4.00	0.14	20	0.00	1.00	15	1.34	0.18	13	1.34	0.18
Dog-directed aggression	**6**	**10.38**	**0.01**	12	1.19	0.24	14	0.99	0.32	11	1.75	0.08
Familiar dog aggression	7	2.67	0.26	10	1.41	0.16	12	0.00	1.00	8	1.34	0.18
Stranger-directed fear	13	2.80	0.25	22	−1.67	0.10	19	−0.35	0.73	16	−0.88	0.38
Nonsocial fear	**12**	**10.17**	**0.01**	18	1.30	0.19	**18**	**−3.00**	**0.003**	17	−0.96	0.34
Dog-directed fear	6	0.95	0.62	10	−1.67	0.10	14	−1.14	0.25	8	−0.95	0.34
Separation-related behavior	13	0.55	0.76	22	0.84	0.40	19	1.63	0.10	16	0.61	0.54
Attachment and attention-seeking	17	1.50	0.47	27	−0.67	0.50	19	1.28	0.20	21	0.08	0.94
**C-BARQ miscellaneous items**												
Escapes	11	0.56	0.76	19	−0.91	0.37	12	0.00	1.00	15	0.07	0.94
Chews inappropriate objects	17	3.65	0.16	**26**	**2.35**	**0.02**	19	1.07	0.29	21	1.33	0.18
Urinates against objects in home	17	0.74	0.69	27	1.10	0.27	19	−1.34	0.18	21	−0.38	0.71
Urinates when left alone	12	1.27	0.53	19	0.65	0.52	18	1.41	0.16	16	0.74	0.46
Defecates when left alone	12	0.29	0.87	19	0.54	0.59	19	1.00	0.32	16	0.58	0.56
Hyperactive, restless, has trouble settling down	17	0.21	0.90	27	−0.91	0.37	19	−0.06	0.95	21	−0.19	0.85
Chases tail/hind end	17	2.63	0.27	27	0.81	0.42	19	1.82	0.07	21	0.63	0.53
Barks persistently when alarmed or excited	17	1.65	0.44	26	1.47	0.14	19	0.75	0.45	21	−0.32	0.75

Bold text shows a statistically significant difference based on timepoint (*p* < 0.05). ^a^ Friedman tests were used to compare C-BARQ scores at two days, two weeks, and four months including dogs with valid data for each time point. ^b^ Wilcoxon signed-rank tests were used to compare C-BARQ scores from the two time points, including dogs with valid data at both points.

## Data Availability

The data presented in this study are available on request from the corresponding author. The data are not publicly available due to data governance arrangements with the animal shelter.

## Supplementary Materials

The following supporting information can be downloaded at: https://www.mdpi.com/article/10.3390/ani12091053/s1, Supplementary Table S1: Dog breed groups.
