# Supplementary material for: Returning a Shelter Dog: The Role of Owner Expectations and Dog Behavior
Source: Animals (Basel). 2022 Apr 19;12(9):1053. doi: 10.3390/ani12091053 (PMC9100056; doi:10.3390/ani12091053)
Supplement: Supplementary file 1 [file animals-12-01053-s001.zip › animals-1668498-supplementary.pdf]

Supplementary Table 1. Dog breed groups.

Breed	Breed group	Not returned		Returned		Total	
		n	%	*n*	%	*n*	%
American Pit Bull Terrier	Pit bull type	1	1.0	-	-	1	0.8
American Staffordshire Terrier	Pit bull type	49	47.6	10	34.5	59	44.7
Australian Cattle Dog	Herding	-	-	1	3.4	1	0.8
Australian Shepherd	Herding	1	1.0	-	-	1	0.8
Beagle	Hound	2	1.9	2	6.9	4	3
Border collie	Herding	1	1.0	-	-	1	0.8
Boxer	Working	4	3.9	-	-	4	3
Bulldog	Non-sporting	1	1.0	-	-	1	0.8
Chihuahua	Toy	1	1.0	-	-	1	0.8
Collie	Herding	1	1.0	-	-	1	0.8
Dachshund	Hound	1	1.0	-	-	1	0.8
German Shepherd	Herding	1	1.0	4	13.8	5	3.8
Golden Retriever	Sporting	1	1.0	-	-	1	0.8
Hound	Hound	15	14.6	5	17.2	20	15.2
Irish Wolfhound	Hound	-	-	1	3.4	1	0.8
Labrador Retriever	Sporting	11	10.7	4	13.8	15	11.4
Lhasa Apso	Non-sporting	1	1.0	-	-	1	0.8
Maltese	Toy	1	1.0	-	-	1	0.8
Pit Bull	Pit bull type	1	1.0	-	-	1	0.8
Retriever	Sporting	1	1.0	1	3.4	2	1.5
Rottweiler	Working	2	1.9	-	-	2	1.5
Shepherd	Herding	4	3.9	-	-	4	3
Shih Tzu	Toy	1	1.0	-	-	1	0.8
Siberian Husky	Working	1	1.0	1	3.4	2	1.5
Spaniel	Sporting	1	1.0	-	-	1	0.8
**Total**		**103**	**100**	**29**	**100**	**132**	**100**
